# Assessing the level of healthcare information technology adoption in the United States: a snapshot

**DOI:** 10.1186/1472-6947-6-1

**Published:** 2006-01-05

**Authors:** Eric G Poon, Ashish K Jha, Melissa Christino, Melissa M Honour, Rushika Fernandopulle, Blackford Middleton, Joseph Newhouse, Lucian Leape, David W Bates, David Blumenthal, Rainu Kaushal

**Affiliations:** 1 Division of General Medicine and Primary Care, Brigham and Women's Hospital, Boston, MA, USA; 2 Clinical Informatics Research and Development, Partners Information Systems, Partners HealthCare, Wellesley, MA, USA; 3 Institute for Health Policy, Massachusetts General Hospital, Boston, MA, USA; 4 Department of Health Policy and Management, Harvard School of Public Health, Boston, MA, USA; 5 Department of Medicine, Harvard Medical School, Boston, MA, USA; 6 Center for Information Technology Leadership, Partners Healthcare, Wellesley, MA USA; 7 Kennedy School of Government, Harvard University, Cambridge, MA, USA

## Abstract

**Background:**

Comprehensive knowledge about the level of healthcare information technology (HIT) adoption in the United States remains limited. We therefore performed a baseline assessment to address this knowledge gap.

**Methods:**

We segmented HIT into eight major stakeholder groups and identified major functionalities that should ideally exist for each, focusing on applications most likely to improve patient safety, quality of care and organizational efficiency. We then conducted a multi-site qualitative study in Boston and Denver by interviewing key informants from each stakeholder group. Interview transcripts were analyzed to assess the level of adoption and to document the major barriers to further adoption. Findings for Boston and Denver were then presented to an expert panel, which was then asked to estimate the national level of adoption using the modified Delphi approach. We measured adoption level in Boston and Denver was graded on Rogers' technology adoption curve by co-investigators. National estimates from our expert panel were expressed as percentages.

**Results:**

Adoption of functionalities with financial benefits far exceeds adoption of those with safety and quality benefits. Despite growing interest to adopt HIT to improve safety and quality, adoption remains limited, especially in the area of ambulatory electronic health records and physician-patient communication. Organizations, particularly physicians' practices, face enormous financial challenges in adopting HIT, and concerns remain about its impact on productivity.

**Conclusion:**

Adoption of HIT is limited and will likely remain slow unless significant financial resources are made available. Policy changes, such as financial incentivesto clinicians to use HIT or pay-for-performance reimbursement, may help health care providers defray upfront investment costs and initial productivity loss.

## Background

Information technology has significant potential to improve patient safety, organizational efficiency, and patient satisfaction in healthcare[1-5]. For example, computerized physician order entry with decision support reduced the serious medication error rate by 55% in one study[6], and some data suggest that electronic medical records can provide a positive return on investment[7,8]. Over the past decade, significant initiatives have been proposed to prompt the adoption of healthcare information technology (HIT)[9-14].

Despite this long standing interest in the adoption of HIT, several studies have suggested that the adoption of HIT in the United States remains limited at least in certain sectors[1,15-17]. Comparison with other industrialized countries further highlights this deficit. For example, 88% of general practitioners in the Netherlands use electronic medical records, but only 17% of their American counterparts do[18]. These statistics point to systemic underinvestment in HIT, which, according to the IOM, has contributed to the quality deficiencies in US healthcare[1].

To estimate the amount of resources required to bridge this investment gap, one needs an accurate assessment of baseline HIT adoption across major stakeholders. However, most prior assessments of HIT adoption have focused on specific IT applications[15,17,19,20] or specific sectors of the healthcare industry[21-23]. Furthermore, while various factors, including the high cost of HIT, resistance from clinicians and vendor immaturity have been cited as barriers against the wide-spread use of specific technologies[24,25], it is unclear how these factors apply across this HIT landscape. We therefore conducted a study to estimate more comprehensively the current level of HIT adoption in healthcare in eight key sectors, and to assess the most significant barriers to further adoption.

## Methods

We adopted a two-phase approach to assess the level of HIT adoption in the US. In phase 1, we performed a detailed analysis of two healthcare markets using in-depth semi-structured interviews with key stakeholders in the two markets. In phase 2, we presented the data collected in phase 1 to a panel of experts to stimulate a discussion about HIT adoption in the US. The experts were then asked to give their estimates for the level of HIT adoption nationally.

### Phase 1: Detailed analysis of 2 healthcare markets

#### Selection of markets and stakeholders

Our market analysis focused on two healthcare markets – Boston, Massachusetts and Denver, Colorado. Boston was selected as one of the two target markets primarily because co-investigators were familiar with this market and had ready access to key informants. Denver was selected as our second target market because several characteristics suggested that it would provide a meaningful contrast to the Boston market, including a lower population density[26], higher penetration of for-profit healthcare institutions[27], and a less prominent presence of teaching hospitals[28] and internally developed HIT systems. Limited resources constrained our in-depth assessment to 2 healthcare markets.

To obtain a comprehensive HIT assessment of the two markets, we identified eight major stakeholders within each market. These 8 stakeholders included: 1) integrated delivery networks, 2) community stand-alone hospitals, 3) skilled nursing facilities and rehabilitation hospitals, 4) physician practices, 5) home health agencies, 6) pharmacies, 7) reference laboratories and 8) third-party payors. These eight stakeholders were selected because of their importance to healthcare consumers. We included payors as a major stakeholder in recognition of their significant effect on the resources that would be available for patient care generally and HIT implementation specifically.

Within each stakeholder group, we used contacts of our expert panel to begin identifying key informants. We then asked our informants to identify other informants knowledgeable about IT adoption in their local area and stakeholder group. We continued to interview informants within a stakeholder group until no further major issues were identified and conceptual saturation was reached.

#### Survey development

In assessing the level of IT adoption in the Boston and Denver markets, we wished to target IT applications with the greatest impact on patient safety, quality of care and organizational efficiency. To identify these applications, we solicited input from a panel of experts convened in January 2003, who also refined and confirmed the framework of our market analyses. The applications selected for in-depth assessment included: 1) electronic results review, 2) computerized physician order entry (CPOE) including electronic prescribing, 3) electronic health record (EHR), 4) claims and eligibility checking, 5) patient-doctor electronic communication, and 6) provider-to-provider electronic communication. For third-party payors, we also targeted incentive-based programs to improve quality of care within our stakeholders. For each functionality domain, we defined a minimum feature and function set based on consensus reached by our expert panel.

Using the list of target HIT applications derived from the expert panel, we developed semi-structured survey instruments for the stakeholders. We used the survey instrument to assess the current level of adoption for each target HIT application in the stakeholder's home institution and plans for future adoption (if any). We also discussed the major barriers and facilitators to adoption of IT in their institution.

#### Data analysis

Interview transcripts were reviewed and analyzed by four co-investigators using the ATLAS.ti™ software package. Illustrative quotes from the informants were also identified. All members of the research team reviewed major findings. The level of adoption for each target HIT application in each stakeholder type was graded on Rogers' technology adoption curve[29] by consensus among the co-investigators. Using Rogers' classification, the co-investigators classified the level of HIT application adoption in each stakeholder as 1) 0–5% (adoption by innovators only), 2) 5–15% (additional adoption by early adoptors), 3) 15–50% (adoption by early majority), 4) 50–85% (adoption by late majority), or 5) 85–100% (widespread adoption). We chose Roger's classification scheme because it has been applied in many settings both within and outside healthcare[30,31].

### Phase 2: national estimates

In the second phase of the project, we attempted to obtain national estimates for the level of HIT adoption. An expert panel was convened for one day in September 2003 for this purpose.

A modified Delphi approach was used to obtain these national estimates. Before the expert panel was convened, all members were asked to provide estimates for the level of adoption nationally in each target HIT application for each stakeholder. Averages of these estimates (expressed as percentages) were calculated and presented back to the experts at the beginning of session. To stimulate discussion, we presented findings from our market analysis in the Boston and Denver areas to the panel. We then asked members of the panel to reach a general consensus about the level of adoption, after which each member independently provided a final estimate for each target HIT application in each stakeholder.

## Results

### Institution demographics

Of the 119 potential informants contacted for telephone interviews, 52 (44%) agreed to be interviewed for phase 1 of our study. The breakdown of these informants in terms of affiliation with a stakeholder group appears in Table 1.

**Table 1:** Informants by Stakeholder and Geographical Location

	Physician Practices	Community Hospitals	Integrated Delivery Networks	SNF/Rehab Hospitals	Home Health Agencies	Pharmacies	Laboratories	Insurance Payors	Misc.	**TOTAL**
Boston	6	3	3	2	2	3	2	2	1	**24**
Denver	4	5	3	3	2	4	1	2	2	**26**
Others*	0	0	0	0	0	0	2	0	0	**2**
**Total**	**10**	**8**	**6**	**5**	**4**	**7**	**5**	**4**	**3**	**52**

SNF = Skilled Nursing Facilities

*2 informants belonged to stakeholders that directly operate in several states

### Characteristics of expert panel

Twelve national experts representing varying interests in the field of medical informatics participated in our expert panel held on 8^th ^Sept 2003. Twelve members of our research team, representing clinical medicine, health services research, informatics, and economics, also participated in the expert panel discussion. Members of the panel have been listed in the acknowledgement section of the manuscript.

### HIT adoption in the Boston and Denver markets

Table 2 illustrates the level of adoption for target HIT applications in the Boston and Denver markets. The level of adoption for each application is graded on Rogers' technology adoption schema[29].

**Table 2:** Adoption of HIT in Boston and Denver

**BOSTON**	Result Viewing	Inpatient EHR	Inpatient CPOE	Ambulatory EHR	Ambulatory CPOE	Electronic Prescribing	Claims/Eligibility	Patient-Doctor Communication
MD Practices	3	--	--	2	2	--	5	2
IDNs	5	2	3	2	1	--	5	2
Stand-alone Hospitals	5	1	3	1	1	--	5	1
SNF/Rehab Hospitals	2	1	1	--	--	--	5	1
Home Health Agencies	1	--	--	2	--	--	5	1
Laboratories	3	--	--	--	--	--	5	1
Pharmacies	--	--	--	--	--	2	5	3
Payors	--	--	--	--	--	--	5	--

**DENVER**	Result Viewing	Inpatient EHR	Inpatient CPOE	Ambulatory EHR	Ambulatory CPOE	Electronic Prescribing	Claims/Eligibility	Patient-Doctor Communication

MD Practices	3	--	--	1	1	--	4	1
IDNs	5	1	2	1	1	--	5	1
Stand-alone Hospitals	5	1	2	2	1	--	5	1
SNF/Rehab Hospitals	2	1	1	--	--	--	4	1
Home Health Agencies	1	--	--	1	--	--	4	2
Laboratories	3			--	--	--	5	1
Pharmacies	--	--	--	--	--	1	5	3
Payors	--	--	--	--	--	--	5	--

Key: (***From adoption curve terminology, Rogers 1983***)

1 = Low adoption by a few innovators (0–5%)

2 = Some adoption by early adopters (5–15%)

3 = Medium adoption by early majority (15–50%)

4 = Common adoption by late majority (50–85%)

5 = Widespread adoption by the rest (85–100%)

In both markets, functionalities to support financial reimbursement were better developed than those used to support safety and quality clinical care. Result viewing was the most widely adopted among the clinical functionalities. Other clinical functionalities were in general only adopted by innovators and early adopters in both markets, with inpatient electronic health records and patient-doctor communication being the least commonly adopted clinical functionalities.

### National estimates for HIT adoption

Table 3 illustrates the estimates for the level of adoption for target HIT applications across the nation. In general, national estimates from the expert panel were in concordance with the findings from the Boston and Denver market analysis.

**Table 3:** National Estimates of HIT Adoption

**National Estimates**	Result Viewing	Inpatient EHR	Inpatient CPOE	Ambulatory EHR	Ambulatory CPOE	Electronic Prescribing	Claims	Eligibility	Patient-Doctor Communication
MD Practices	24%	--	--	9%	5%	--	79%	11%	6%
IDNs	61%	20%	15%	13%	10%	--	90%	28%	8%
Stand-alone Hospitals	55%	12%	9%	7%	6%	--	85%	19%	4%
SNF/Rehab Hospitals	8%	1%	1%	--	--	--	77%	17%	1%
Home Health Agencies	6%	--	--	5%	--	--	73%	16%	2%
Laboratories	86%	--	--	--	--	--	90%	47%	6%
Pharmacies	--	--	--	--	--	5%	93%	76%	26%
Payors	--	--	--	--	--	--	94%	86%	--

### Barriers and facilitators to the adoption of HIT

We describe here the major barriers and facilitators to the adoption of HIT within each of our stakeholder groups. Where appropriate, we have used representative quotes from our informants to illustrate the major issues they face.

#### Integrated delivery networks (IDNs)

IDNs came into existence in the 1990's primarily through the merger of hospitals[32]. These networks represent aggregations of acute care hospitals, rehabilitation hospitals, and ambulatory care practices. Some even provide nursing home or home care services. Since many of the entities that comprise an IDN had existing legacy information systems before the mergers, integrating clinical data across various institutions remains a work in progress. While most individual institutions have access to results performed locally, access to results performed elsewhere in the network is not guaranteed, especially in the Denver market.

As several forms of HIT may require significant upfront investments and may cause significant disruptions in clinicians' workflow at least initially, most IDNs have had to make choices about the types of HIT to adopt. In the words of one CIO at an IDN:

"*Result report has to be done early and quickly. Provider order entry is just good from a business quality standpoint. You may put in other things that enhance workflow, revenue cycle systems billing capture. EMR is absolutely the last thing you want to do because it takes amounts of physician time to do the work*."

As suggested by this particular CIO, the use of inpatient CPOE systems remains limited to early adopters, although with the endorsement of CPOE as one of the three quality markers for hospitals, many hospitals are actively considering its adoption. This is evident in the national estimates provided by our expert panel, with only 15% of IDNs having adopted CPOE systems. The Boston market emerged as slightly more advanced in terms of CPOE adoption, as the success of CPOE implementation at various academic medical centers has encouraged smaller hospitals within the IDN to adopt this innovation.

Adoption of other advanced clinical information systems, such as ambulatory EHR, ambulatory CPOE, and inpatient EHR, remains limited to either select innovators or early adopters. Truly innovative systems such as patient-doctor communication via secure email remain experimental.

Financial information systems are more widely available than clinical information systems in both markets studied. HIPAA legislation was frequently cited as an enabler, as several institutions had recently expended significant efforts to comply with the mandated data standards. Notably, IDNs and payors in the Boston area have come together to form the New England Healthcare Electronic Network (NEHEN)[33] to further streamline and standardize the submission and adjudication of payment claims. This arrangement has resulted in significant savings for both provider institutions and payors.

#### Stand-alone hospitals

While stand-alone hospitals do not face the issue of data integration encountered by IDNs, they face similar issues in the adoption of clinical information systems with regards to costs and clinician resistance. In addition, since most physicians working in community hospitals are not employees of the hospital and can admit their patients to competing hospitals, hospitals often find it hard to the enforce the use of new HIT applications such as CPOE. Training is also problematic, as many of these physicians spend only a fraction of their time in the hospital and are not motivated to find time to attend training sessions. Overall, these smaller hospitals currently face significant challenges in the adoption of HIT in terms of the lack of capital and clinicians' enthusiasm for this new technology, as exemplified by the following comment:

"*It is a smaller facility, so money is not there, nor is there a huge demand for IT. People are not beating on the door asking for that stuff*."

Stand-alone hospitals tend to deploy systems made by one vendor to minimize the cost of cross-system integration. These systems tend to be 'vanilla' in functionality, offering a basic bed control system, a pharmacy system, laboratory information system with result viewer, and perhaps a non-physician order entry system. Again, advanced systems such as inpatient clinical documentation and patient-doctor electronic communication remain the purview of a few pioneering hospitals. We noted, however, that many community hospitals are seriously considering the deployment of CPOE systems, often in response to the endorsement of CPOE as a patient safety marker by the Leapfrog group.

#### Skilled nursing facilities/rehabilitation hospitals

Skilled nursing facilities and rehabilitation hospitals stood out as stakeholders that are most behind in the adoption of HIT. In one facility, the informant told us that they "[had] *a very old DOS-based system*." Many of these facilities currently use HIT in a limited fashion, mostly for bed control and billing purposes and not for clinical care. EHRs and CPOE systems are rare. Internet access usually is by phone lines, and review of test result typically occurs on paper. Provision of electronic communication between clinicians and patients or their families was almost unheard of.

Despite the generally grim picture painted by our informants, they identified potential leverage points. First, since the advent of diagnostic-related group reimbursement, acute care hospitals have been financially motivated to discharge patients early. Some acute care facilities have therefore invested directly in HIT systems in chronic care facilities so that these chronic care facilities can be better equipped to admit sicker patients discharged from acute care facilities. Second, chronic care facilities, particularly nursing homes, are heavily regulated and many are required to file detailed reports to payors. In select facilities that are using HIT to fulfill this filing requirement, our informants identified opportunities to develop an electronic health record that can serve regulatory and clinical needs concurrently.

#### Physician practices

While physician clinics vary in size, most of the clinics in the US have between 1 and 4 physicians[34]. Most practices therefore are run as small independent businesses, with many reporting declining incomes. For these practices, productivity and billing are their top concerns. Furthermore, such practices typically have little access to capital. The following comments express the sentiment typical of our physician practice informants:

• *Question: "What's your budget on IT last year?" Practice Manager at a physician practice: "$1200"*

• "*When you are faced with the pressures that are put upon us in our crazy health care system, I think that you get into survival mode. As much as [we] would like to get involved [in improving quality with information technology], we just can't plain afford it at this point*."

As with other stakeholders, HIT is used extensively to manage billing and scheduling of patients. However, for clinical purposes, physicians in office practices still rely on the paper chart. While there is significant interest in deploying EHRs and electronic prescribing, many physicians are reluctant to move ahead with the technology. There are several reasons for this: first, the significant investments in EHRs have to be borne by physicians, while the benefits, including improved clinical outcomes and fewer adverse events, accrue to patients and payers of health insurance premiums[8]. In addition, improved clinical outcomes generally do not allow clinicians to command higher reimbursement. Second, there are significant concerns about the impact of new technology on productivity especially at the beginning. Third, there is a perception that failed implementations of EHRs in small practices significantly outnumber successes, and practices are very reluctant to take on this risk without a more stable vendor industry climate.

#### Pharmacies

We were pleasantly surprised to find that many pharmacy chains have developed relatively advanced HIT systems. Transactions with pharmacy benefit management (PBM) programs to check for patients' eligibility for medications occur electronically on a routine basis, and the cost of building this infrastructure is accepted as 'the cost of doing business'. Since many PBM programs offer some degree of decision support to check for drug-drug interactions, that benefit is passed on to the patient through the pharmacy. Some of the more pioneering pharmacy chains stores have already developed sophisticated on-line communication systems through their websites to provide information on medications and to allow patients to renew their medications on-line.

Connectivity between pharmacies and other healthcare institutions remains limited, however. Electronic prescribing is only beginning to be adopted by the early adopters of this technology. Several barriers remain: first, concerns about cost and workflow remain for a good proportion of physician practices; second, legislative barriers still need to be addressed – one informant in Denver described the series of road blocks her institution encountered when they tried to convince the Colorado state legislature to allow the electronic transmission of prescriptions.

#### Home health agencies

The lack of affordable mobile high-speed networks poses a challenge for the deployment of HIT in home health agencies. Current efforts are mostly focused on fulfilling billing and reporting needs. Of the HIT that has been deployed, little focuses on patient safety or quality. Communication between home health providers and primary care physicians remain a challenge, and there appear to be few drivers for the integration of home health care HIT systems with primary care systems.

Home monitoring, however, holds promise. In Colorado, where there is a shortage of nurses and existing nurses need to spend a higher proportion of their time driving, there has been growing deployment of remote vital-sign monitoring systems. Our informants believe that these systems allow visiting nurses to safely reduce the number of face-to-face visits, and thus increase the number of home patients a visiting nurse can be responsible for concurrently. In the Boston area, however, where patients tend to live close to metropolitan areas, the development of these remote monitoring systems appears to be more nascent.

#### Laboratories

While the use of HIT within reference laboratories to track specimens and to store results appears fairly advanced, use of electronic means to report test results back to providers appears mixed. Although major reference laboratories have set up websites to provide secure result reporting to providers to review test results, integration of the results into electronic medical records remains onerous. Most reference laboratories have not built interfaces with different EHRs and hospital information systems. As a result, much of result reporting is still on paper.

#### Third-party payors

Previous measure to control medical expense, such as capitation, prior authorization and financial withholds have angered providers and patients alike. It is against this backdrop that payors have started to look at approaches that better align the interests of the patient and the payors, and pay-for-quality incentives schemes have emerged as an important lever. However, for these schemes to be successfully implemented, systems must be in place to measure quality and providers need better systems to improve quality of care. Increasingly, payors believe that clinical HIT systems can play these roles. Innovative programs such as Bridges-to-Excellence sponsored by General Electric and Verizon are experimenting with ways to provide financial incentives to physicians for purchasing HIT systems to improve quality of care[35].

Other areas of innovation include efforts by Blue-Cross-Blue-Shield plans to reimburse physicians for providing clinical care on-line, efforts to standardize exchange of billing data across stakeholders, and efforts to provide details of pharmacy benefits and medication history for each patient to the provider at the point of prescribing. While these early initiatives have yet to be diffused, many of our informants from various stakeholders groups saw them as potential avenues to improve the diffusion of HIT.

## Discussion

This study suggests that HIT adoption remains limited and variable across key stakeholders. Use of HIT appears to be predominantly driven by financial functions, as reflected by the relatively ubiquitous use of electronic claims submission checking. While there is increased interest in the adoption of technology to improve the quality of care, significant adoption challenges remain, particularly in the area of EHRs and physician-patient communication.

Organizations face enormous financial challenges in adopting HIT. Our informants consistently discussed the large upfront investments necessary to deploy these systems, and only a small fraction of institutions, predominantly large institutions such as IDNs, could afford comprehensive versions of these systems. Ironically, the low penetration of HIT may itself contribute to its high cost, as each vendor must charge its small customer base a relatively high fee in order to recuperate research and development costs. These fees might put HIT out of the financial reach of small organizations, particularly small physician practices.

Misalignment of incentives is an important barrier to HIT adoption. Initial capital expenditures of HIT are high and payers do not directly compensate institutions or providers for its use, or for the resulting higher quality and safer care. This coupled with a hard-to-assess return on investment makes it difficult for institutions to risk making even necessary purchases[12]. Decision makers tend to invest in areas that can readily be seen to be directly financially beneficial to the institution, which under the current reimbursement scheme are areas like MRI scanners and new buildings rather than IT infrastructure. While financial benefits can be realized from network investment, these benefits are seen across many IT cost centers and are hard to measure.

Organizations face enormous financial challenges in adopting HIT. Our informants consistently discussed the large upfront investments necessary to deploy these systems, and only a small fraction of institutions, predominantly large institutions such as IDNs, could afford these systems. Small organizations, particularly small physician practices, find it hard to purchase and maintain these systems. These practices are highly risk-averse and are justifiably fearful about the possibility of implementation failures and are therefore less likely to take the initiative to deploy these systems. While these institutions are small, they deliver the bulk of medical care in the US[34]. Therefore, their financial barriers to the implementation of HIT have significant implications for the nation's quality improvement agenda in healthcare.

Apart from the issues of cost, significant concerns remain regarding the impact of HIT initiatives on productivity. Several institutions we interviewed cited the up-front loss of productivity during the transition from paper-based to computer-based systems. When the income of health-care providers is directly tied to their productivity but not to their quality, this resistance to change could be particularly difficult to overcome in the era of decreasing reimbursement. However, research seems to suggest that the negative impact of EHR implementation on productivity is modest and may diminish over time[36,37]. Such discordance between provider perception in the community and findings from these recent studies performed at academic institutions may be attributable to several factors. First, usability and clinical decision support among vendor products vary significantly. Second, fewer resources may be available to train physicians in the community setting. Third, many small practices may not be able to afford the fees vendors might charge to customize the HIT products to fit the local workflow. Therefore, future research, possibly in collaboration with vendors, needs to focus on ways to design applications that would be intuitive to even new users and adaptable to the different workflow patterns in small practices.

While other industries, such as the financial and computer industries, have long established uniform standards for the interchange and ordering of parts and data, health care has lagged behind. The lack of data standards makes it difficult to manage the myriad of existing homegrown and vendor systems each organization might own. This, in turn, makes it very costly to invest in more advanced HIT capabilities. The lack of interoperability between different data sources also undermines the usefulness of HIT to the clinician, who may be asked to use a variety of paper-based and electronic methods to retrieve and enter data even for the same patient. The challenges associated with using these non-interoperable HIT systems may negatively impact workflow and productivity, which in turn contribute to clinicians' resistance to adopt these systems[24].

Within the markets we studied, the use of HIT varied considerably across the stakeholders. For example, while several IDNs in both Boston and Denver are making major investments in HIT, most nursing homes and rehabilitation hospitals lag behind significantly. Small physician practices are highly risk-averse and are justifiably fearful about the possibility of implementation failures and are therefore less likely to deploy HIT. This variation in the use of HIT across stakeholders is noteworthy from several standpoints. Since patients often transition from acute-care settings to non-acute care settings, the improvement in quality gained through HIT investments in acute-care hospitals may be attenuated by the under-investment in chronic care institutions and physician practices. While these non-acute facilities are often small in size, they deliver the bulk of medical care in the US[34]. Therefore, the financial barriers that they face have significant implications for the nation's quality improvement agenda in healthcare. Furthermore, our informants at chronic care institutions and many of the physician practices indicated that this underinvestment in HIT would continue at these organizations. If that is indeed the case, the investment gap between acute care and non-acute care facilities would likely widen, potentially leading to greater disparities in quality. From a healthcare policy perspective, our findings point towards the urgent need to understand ways to overcome the barriers to diffusing HIT in chronic-care facilities and small physician practices.

The findings of this study should be interpreted in light of its limitations. First, we acknowledge that the qualitative methods used in this study may yield less precision than quantitative methods, although collecting quantitative data in all the stakeholders covered in our study would be very difficult. Second, while there is good agreement between the estimates of HIT adoption in the two markets and the estimates provided by the expert panel, we acknowledge that our expert panel was probably influenced by the market analysis we performed. Third, the selection of Boston and Denver were based our access to contacts in those markets, and not all of the potential informants we contacted participated in the interviews. Therefore, the results of our qualitative analysis might have been subject to selection and responder biases. Both forms of biases would likely have caused the research team to over-estimate the level of HIT adoption, and the actual levels of adoption may be even lower. Fourth, the national adoption estimates derived from our expert panel were likely influenced by their personal biases. Fifth, our limited resources prevented us from studying in depth the adoption of HIT in more than two markets, and regional variations in HIT adoption would have been difficult to discern.

In summary, HIT adoption in the US remains in its infancy, and the significant disparities in adoption among key stakeholders will likely worsen unless incentives for HIT adoption can be realigned to reward quality rather than quantity of care. Several levers, including quality-based financial incentives and adoption of standards by payors, will likely represent significant facilitators of this process. Despite the daunting challenges that lie ahead, US policy makers and health care institutions should take heart in the fact that other industrialized nations have been successfully deploying HIT to improve the quality of care[18,38].

## Competing interests

The author(s) declare that they have no competing interests.

## Authors' contributions

• EGP, AKJ, RF, BM, JN, LL, DWB, DB and RK participated in the conception of the design.

• EGP, AKJ, MC, RK performed the semi-structured interviews.

• EGP, AKJ, MC, MMM performed the qualitative analysis.

• EGP, AKJ, MC, RF, DWB, DB and RK organized the expert panel group, collected, and analyzed adoption estimates from members from the panel.

• EGP drafted the manuscript, which was revised and approved by all co-authors.

## Pre-publication history

The pre-publication history for this paper can be accessed here:


